# Online faculty development in low- and middle-income countries for health professions educators: a rapid realist review

**DOI:** 10.1186/s12960-022-00711-6

**Published:** 2022-01-29

**Authors:** Lianne Keiller, Champion Nyoni, Chantel van Wyk

**Affiliations:** 1 Division of Health Sciences Education, Faculty of Health Sciences, University of the Free State, 205 Nelson Mandela Drive, Park West, Bloemfontein, 9301 Free State South Africa; 2 School of Nursing, Faculty of Health Sciences, University of the Free State, 205 Nelson Mandela Drive, Park West, Bloemfontein, 9301 South Africa

**Keywords:** Health professions education, Online faculty development, Faculty development, Community of practice, Educational design research, Conjecture mapping, Low-and middle-income countries

## Abstract

**Background:**

Health professions educators require support to develop teaching and learning, research, educational leadership, and administrative skills to strengthen their higher education role through faculty development initiatives. Where administration has pursued face-to-face and online faculty development initiatives, results have positively influenced health professions educators. There is limited evidence demonstrating how online faculty development works for health professions educators in low- and middle-income countries who engage in online health professions education (HPE) faculty development.

**Methods:**

A Conjecture Map for online HPE faculty development courses identified candidate theories for a rapid realist review. The Conjecture Map and candidate theories, Community of Inquiry and the Conversational Framework guided the development of search terms and analysis for this review. Three searches using EbscoHost databases yielded 1030 abstracts. A primary and secondary research team participated in a multi-reviewer blinded process in assessing abstracts, selecting full-text articles, and data extraction. The primary research team analysed eight articles for this rapid realist review to answer the research question: How do online HPE faculty development courses work, or not work, in low- and middle-income countries? Data were analysed and mapped to the initial Conjecture Map and the research question.

**Results:**

The research references US-based organisations forming partnerships with low- and middle-income countries, and who provide funding for online HPE faculty development initiatives. These initiatives design courses that facilitate learning through engagement from which participants report beneficial outcomes of professional and career development. The review does not clarify if the reported outcomes are generalisable for facilitators from low-and middle-income countries. The findings of this review demonstrate the role of a community of practice as the dominant mechanism through which the outcomes are achieved, based on a design that incorporates six triggering events. The design aligns the triggering events with the three categories of the Community of Inquiry—a theory for designing online learning environments.

**Conclusion:**

Health professions educators in low- and middle-income countries can develop professional and interpersonal skills through a well-designed, specifically constructed online community that prioritises active discussion.

**Supplementary Information:**

The online version contains supplementary material available at 10.1186/s12960-022-00711-6.

## Contributions to the literature

- Health Professions Education faculty development research primarily represents face-to-face, institution-based initiatives situated in middle and high-income countries. The same is true for online faculty development. This influences the generalisabilty of evidence for low- and middle-income countries.
- Our study utilises an innovative approach to addressing this issue with a rapid realist review that uses Conjecture Mapping as the basis for, and output of the research. This innovation demonstrates the possibilities of combining the theoretical output of a review with the practical application of education design research.
- The findings in this study highlight the importance of local, contextually relevant, theoretically sound research.

## Introduction

Faculty development is an inherently complex concept due to the nature of the interventions and the recipient context's variability, approach to delivery and purpose. Many literature, narrative and scoping reviews related to faculty development report positive programme outcomes [1] without expanding on the mechanism's complexity and operational standing to achieve these [1–4]. One of these reviews specifically called for further research into online faculty development to determine what works, for whom and in what circumstances [3]. This call reinforces the longstanding need to investigate the complexities inherent in online faculty development, catalysed in 2020/2021 by face-to-face facilitation restrictions during the COVID-19 pandemic.

Faculty development has evolved rapidly over the last 30 years by adapting to changes in the environment, educational theory and technology [5]. Globally, the literature reports that faculty development opportunities are usually institution-specific, in-person programmes or workshops for non-degree purposes, or delivered as part of a postgraduate qualification [1, 4, 6, 7]. Faculty development practitioners generally claim these faculty development opportunities positively impact health professions educators [1, 4, 8] who require support to develop their teaching and learning, research, educational leadership, and administration skills. These skills are part of the health professions educators' practice. Their development strengthens their role in higher education, especially in low- and middle-income countries, where professional development opportunities are scarce [6].

The current situation within low and middle income countries limits opportunities for professional development of health professions educators in practice. At a national scale, there is limited funding for human resource development, where other competing national priorities such a healthcare service delivery take precedence [9]. Health professions educators who happen to have opportunities for professional development are normally engaged in specific clinical skills development and not necessarily focused on approaches to teaching and learning, research and educational leadership. Arguably, due to limited resources within the low- and middle-income countries—there is limited numbers of professionals with expertise to develop health professions educators. This situation creates an opportunistic approach towards faculty development of health professions educators in the low- and middle-income countries through institution-based piece-meal programmes that may not be transferrable. These programmes may not be underpinned by educational theory limiting approaches to scientifically evaluate their influence within low- and middle-income countries. Theoretically sound online faculty development has the potential to positively impact the practice of health professions educators. Educational design literature describes frameworks and guidelines grounded in theory for designing and delivering online teaching and learning [10] that creates an opportunity for deep and meaningful collaborative learning. Three of these frameworks are Community of Inquiry, Conversational framework and Community of Practice. The Community of Inquiry, as an example, has three transactional factors that intersect to enhance the effectiveness of learning in blended and online learning environments. Two of these factors, namely, cognitive presence, where learners construct meaning through sustained reflection and discourse, and teacher presence, where the teacher or facilitator meaningfully facilitates course design with collaborative intent, relate directly to the learning environment design [11]. Furthermore, the Conversational Framework [10] provides educators with a theoretical approach to teaching and learning in a technology-driven society by framing the approach to teaching as an interactive dialogue. Utilising multiple learning theories, the Conversational Framework presents learning and teaching as an iterative process of generating and modulating concepts between the teacher, learner and the environment [10]. A realist review [12] found the Conversational Framework to be prevalent in internet-based medical education. In a Community of Practice (CoP), group participatory learning takes place and technology offers more people to learn together through the use of online communities [13]. In this paper, the authors argue that a Community of Practice (CoP) underpinned by the Conversational Framework and Community of Inquiry is crucial for the positive outcomes of an online faculty development course for health professions educators in low- and middle-income countries.

First developed as a concept linked to social learning, CoPs consist of three dimensions; a joint enterprise, mutual engagement and a shared repertoire of communal resources focused on organisational learning [14]. Two decades after Wenger's seminal work on COPs was published, Li and colleagues proposed a set of characteristics for CoPs within the health professions sector [15]. These characteristics are a context that supports formal and informal interaction, fosters a sense of belonging, and emphases learning and sharing knowledge [15]. Conversely, these characteristics align with the theoretical assertions of understanding online learning through a Community of Inquiry lens [11]. The social, cognitive and teaching presence described in the Community of Inquiry [11] aligns with the interaction between peers and between facilitators/peers, social learning and knowledge sharing. However, the evidence for improved practice through CoPs has been limited to initiatives with explicit efforts aimed at developing communities in face-to-face or blended community environments [4, 16, 17] middle- and high-income settings.

This paper presents a synthesis of literature with an explanatory focus aimed to unpack the mechanisms of how online faculty development work (or why they fail) in low- and middle-income countries by establishing causal relationships necessary to understand underlying mechanisms and the context in which those relationships occur.

## Objectives of the review

The objective of this review was to establish the context, mechanism, and outcome (CMO) of online health professions education faculty development in low- and middle-income countries. The review question was: How do online health professions education faculty development programmes work, or not work, in low- and middle-income countries?

## Methods

### Rapid realist review

This study adopted a rapid realist review approach [18]. This approach focuses on explaining the question under review in a time-sensitive manner instead of the lengthy, consultative approach in definitive theory development [18, 19] traditionally produced in a realist review. This methodological approach addresses the call for more rigorous research related to educational technology and design research that moves beyond the "What works?” questions to the deeper, theoretical and knowledge generation required for solving academic problems [20]. The RAMESES publication standard [21] guided the structuring of this paper. A primary research team (the authors) and a secondary research team which was purposively selected based on their experience and expertise in faculty development in low- and middle-income countries with professional backgrounds in medical sciences, nursing and public health—contributed to this review.

### Conjecture mapping

**Fig. 1:**
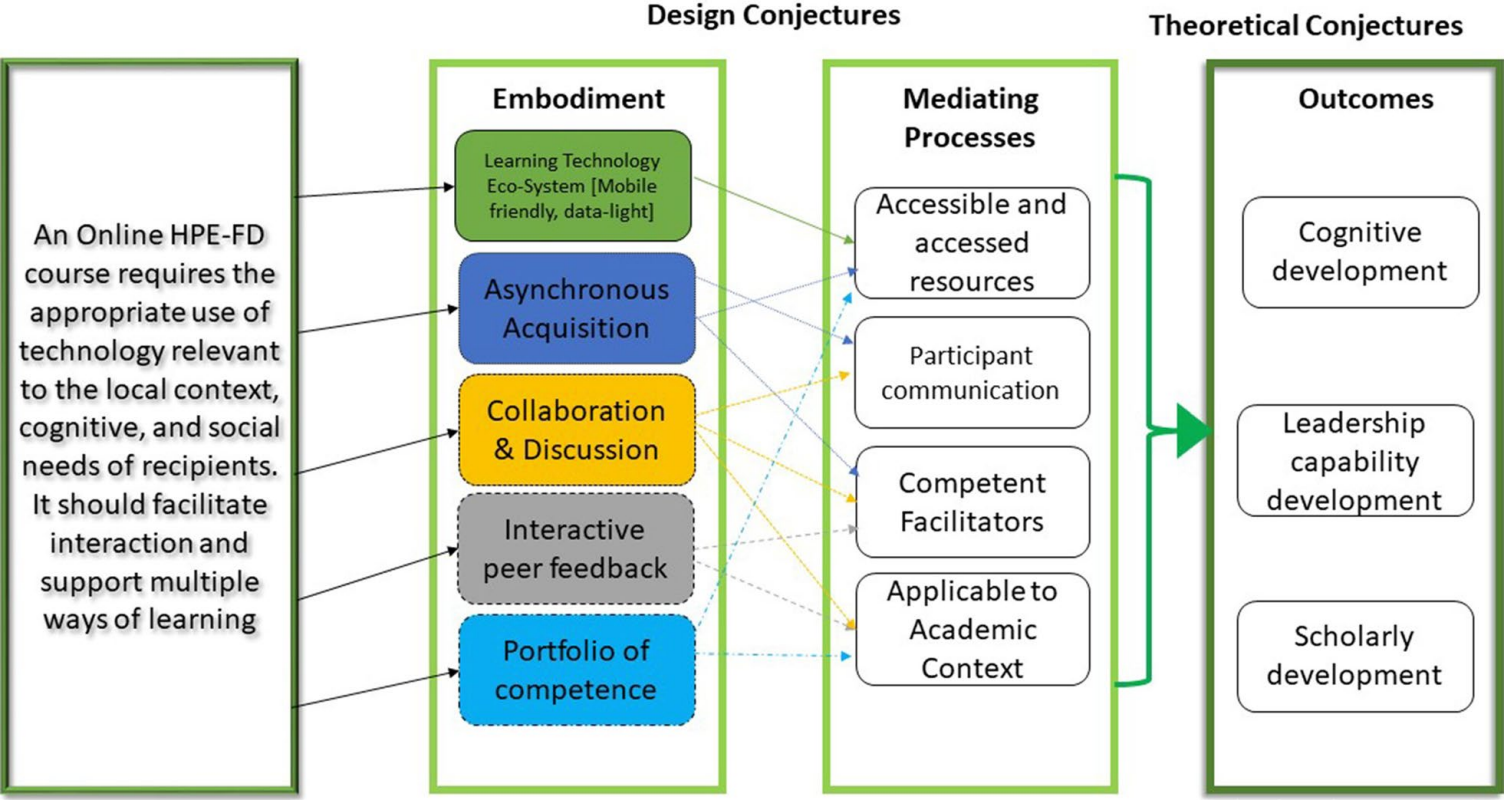
Preliminary conjecture map

The researchers initiated the rapid realist review through the development of a Conjecture Map. A conjecture map is a research planning and organisation tool, and, in this study, it was used as a way to demonstrate the theoretical nature of online health professions education faculty development design. A conjecture reflects a hypothesis in which the researcher assumes education takes place, with embodiment reflecting tools, activities, materials, participant structures and discursive practices necessary to support the conjecture. These are then influenced by necessary conditions—known as mediating factors leading to specified outcomes. The approach is underpinned by the pedagogical and design theories of the Conversational Framework [10] and Community of Inquiry [11]. The conjecture, or hypothesis of how the proposed outcomes of an online faculty development course are achieved, served as a proposed programme theory to align with the realist review methodology [22] (Fig. 1).

This Conjecture Map provided the basis for identifying the search terms, developing the data extraction tool, and analysing selected articles. To our knowledge, this has been the first rapid realist review to apply this approach.

### Search string

The primary research team identified the Population, Intervention, Context and Outcome [23] (PICO), and inclusion/exclusion criteria relevant to this study. The population referred to health professions educators OR medical educators OR medical teachers OR health professions academic faculty. The terms are interchangeably used for professionals within a medical or health sciences field who are involved in academic teaching–learning. The interventions included were online faculty development OR technology-mediated health professions education. The context and outcomes specific to the search were low- and middle-income countries and professional development programmes OR faculty development.

The PICO criteria served as search terms applied to EBSCOhost databases, applying the search string to the following databases: Academic Search, Academic Search Ultimate, Africa-Wide Information, Business Source Ultimate, CAB Abstracts, CINAHL with Full Text, Communication & Mass Media Complete, ERIC, GreenFILE, Health Source: Consumer Edition, Health Source: Nursing/Academic Edition, Humanities Source Ultimate, OpenDissertations, APA PsycArticles, APA PsycInfo, Sociology Source Ultimate, MasterFILE Premier, MEDLINE with Full Text, and Scopus. The search process was conducted in 3 phases (Additional file 1).

### Selection of the literature

The following inclusion and exclusion criteria guided the selection of the articles for this review:

Inclusion criteria:

- Publication between 1 January 2010 and 1 August 2020
- Full-text articles, commentaries and editorials published in peer-reviewed journals.
- Research reporting on online faculty development initiatives in HPE/medical education.
- Research conducted in low- and middle-income countries focused on online HPE faculty development.
- Faculty development initiatives delivered using a fully or partially online platform

Exclusion criteria:

- Studies presenting research on undergraduate student participants.
- Faculty development initiatives that do not include an online component.
- Reviews and other non-primary research

### Search results

Three searches identified 1 836 citations for abstract review. One member of the primary research team manually removed all duplicates after automatic deduplication by the librarian assisting with the search. Titles and abstracts (*n* = 1030) were evaluated to confirm the primary studies' compliance with the inclusion and exclusion criteria. Studies conducted in countries identified as low and middle income by the World Bank in 2020 were considered for inclusion [24]. The primary research team, after discussion and consensus, selected fifty abstracts for a full-text evaluation. One of the abstracts was not available to the researchers.

**Fig. 2:**
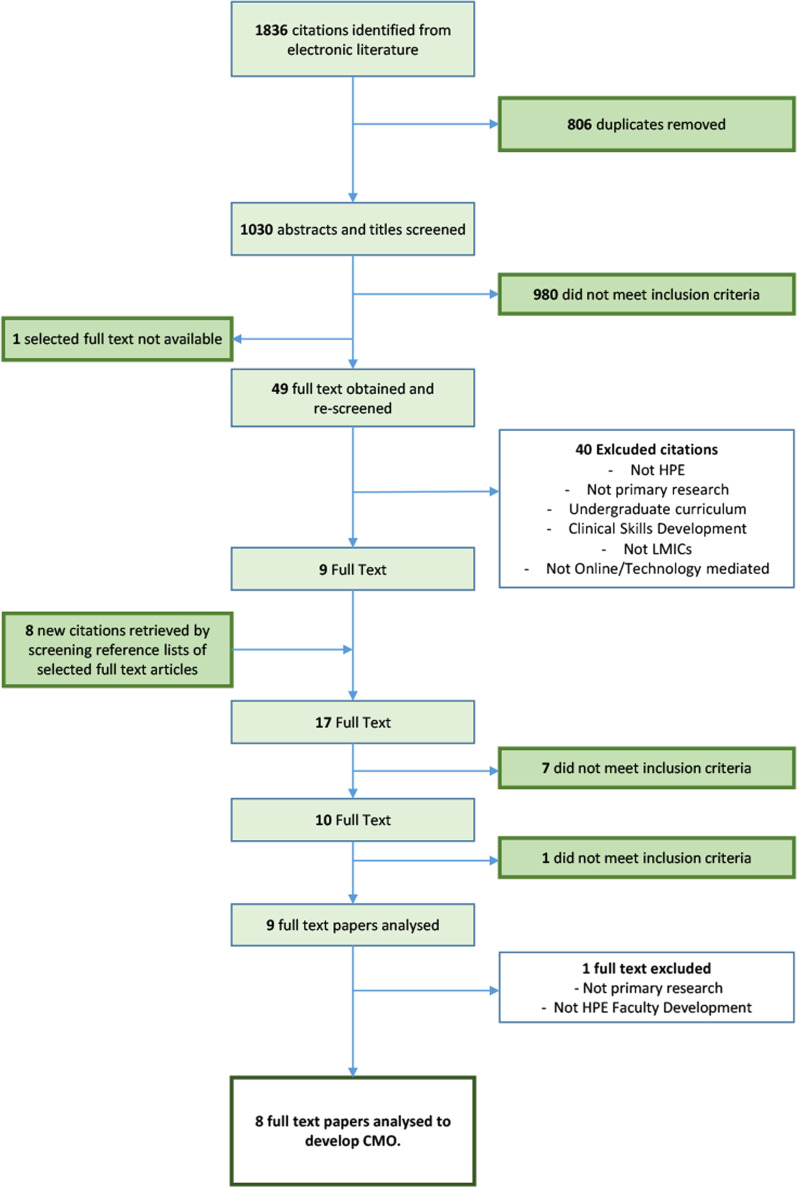
PRISMA flowchart

A secondary research team member and two primary researchers used a custom-designed tool (Additional file 2) to evaluate 49 full-text articles for eligibility in the review. The researchers, through a consensus-building discussion, discarded articles that did not meet the inclusion criteria. The team selected nine full-text articles for inclusion [25–33], reviewing the reference list for additional literature. However, the search did not yield additional articles. Upon further analysis, one article [25] did not meet the criteria, eliminating it from the final analysis (Fig. 2).

### Data extraction

Two members of the primary research team piloted the data extraction tool (Additional file 2). The first author and two secondary research team members independently extracted data from the selected full text. Data extracted included the location, facilitator and participant descriptors and recruitment, intervention, tools and materials used, outcomes of the intervention and recommendations. The first author consolidated all extracted data in a single document for analysis by the primary research team.

### Analysis and synthesis process

The researchers adopted a phased analysis approach. In the first phase, each article was numerically coded for the synthesis process. The team summarised each of the selected articles to stipulate the intervention's geographical location, context, mechanism, outcomes and theoretical justification for the faculty development programmes' success or failure. The second phase consisted of an inductive thematic analysis of the CMO classifications from phase one. Finally, the researchers mapped the themes identified in the second phase of the analysis to the original Conjecture Map and candidate theories. The analysis included an inductive thematic analysis to categorise the data, map categories against candidate theories and create a draft narrative analysis of the Context, Mechanism and Outcomes. The findings of the analysis are described according to these three phases.

## Main findings

### Context, mechanism and outcome

The CMO analysis yielded the following results (Table 1).

**Table 1:** CMO analysis

Code	Title	Country	Context	Mechanism (Resource)	Mechanism (Reasoning/Why)	Outcome
1	Ahmed (2013) Tailoring online faculty development programmes: overcoming faculty resistance Reference: [23]	Egypt	Invited Expert Ophthalmology & Vascular surgery clinician and faculty facilitators and participants	Participant selects learning needs via email invitation, custom designed online course, Assignments and expert-led discussions, f2f workshop	External expert involvement and self-directed learning improved participation and satisfaction, decreased resistance	Increased participation, Participant satisfaction
2​	Anshu, Sharma, Burdick & Singh (2010) Group Dynamics and Social Interaction in a South Asian Online Learning Forum for Faculty Development of Medical Teachers Reference: [24]	India	Fellows & Fellow-moderators in HPE fellowship programme and facilitators for programme	Email listServe discussion on topic selected by participants and facilitated by near-peers with faculty mentoring of the moderators	community of inquiry (Social Presence) supported by involvement of moderators supports learner-centred design and modification of curriculum activities	High level of social presence supporting cognitive and teacher presence for promoting ongoing discussion to engage with topic
3​	Dongre, Chacko, Banu, Bhandary, Sahasrubudhe, Philip & Deshmukh (2010) Online Capacity-Building Program on “Analysis of Data” for Medical Educators in the South Asia Region: A Qualitative Exploration of our Experience Reference: [25]	India, Nepal, Malaysia	Fellows & Fellow-moderators in HPE fellowship programme and facilitators for programme	Email listServe discussion on research methods to support Fellows in conducting HPE innovation research	Adult learning principles that supports a flexible, reactive learning environment. ​	Discussion provided support in study design, implementation and analysis plans and production of technically robust research work. Email discussion highlighted learning needs and helped shape the learning outcomes for the group.​
4​	Frantz, Bezuidenhout, Burch, Mthembu, Rowe, Tan, Van Wyk & Van Heerden (2015) The impact of a faculty development programme for health professions educators in sub-Saharan Africa: an archival study Reference: [26]	South Africa	Fellows in HPE fellowship programme and HPE expert facilitators for programme	2 year blended fellowship programme. 3 f2f sessions with workshops on scholarship, project management, teaching and leadership. Online/distance learning sessions	A broad, context-sensitive knowledge of HPE as well as excellent teaching and research skills to run such programmes facilitates the outcomes identified meeting Kirkpatrick Model of evaluation levels	(1) belonging to a community of practice, (2) personal development, (3) professional development, (4) use of tools and strategies for project management and/or advancement, and (5) capacity development
5​	Ladhani, Chatwal, Vyas, Iqbal, Tan & Diserens (2011) Online role-playing for faculty development Reference: [27]	Pakistan India Malaysia USA	Fellows & Fellow-moderators in HPE fellowship programme and facilitators for programme	Role-playing scenario sent via email listServe focused on CBME as theoretical topic. Participants required to use available materials on the topic to respond to a fictitious Dean's letter as various stakeholders	Adult learning and online role-playing: Encouraged distributed participation among a diverse population, keeping participation and interest high due to the role-playing approach used	The discussion helped to clarify concepts related to CBME, and generated many themes; 10 models of CBME from various countries. The active participation and high level of engagement had an impact on subsequent online discussions on the list server. role-playing was picked up and used in the virtual session discussions by the fellows in one of FAIMER’s four regional institutes. This re-use of the learning strategy by other fellows (health professions faculty members) is probably the best evidence that this group finds role-playing to be effective and 6enjoyable for the participants
6​	Naeem & Khan (2019) Stuck in the blend: Challenges faced by students enrolled in blended programs of Masters in Health Professions Education Reference: [28]	Pakistan​	Masters (HPE) students and internal/external teaching faculty	Blended MHPE programme using both f2f and online activities	Constructivist and collaborative learning approach used. Community of inquiry used to analyse. Issues identified, where learner presence was negatively influenced by issues related to self-regulation and as a result of previous experience as spoon-fed learners. Infrastructure and internet access negatively influenced student experience and participation	Students struggled to develop autonomy, were overloaded on a cognitive level and required facilitators to actively manage group dynamics with a group who struggled with time management as a result of work-requirements. Lack of support from institution also negatively influenced student participation and success of the programme.​
7​	Thakurdesai, Ghosh, Menon, Sahoo, Tripathi, Harshe & Andrade (2018) Electronic journal clubs for capacity building: A case study in psychiatry as a model for medical disciplines in developing countries Reference: [29]	India	Psychiatrists (post-graduate students, recent graduates, academic faculty and clinicians)	Electronic journal club for discussion of articles, training on manuscript review and scholarly analysis and writing	Can be replicated in other developing countries for more efficient manpower development and capacity building in academic medicine. The availability of active, committed, competent, and experienced facilitators is needed	Improved analytical and as well as writing skills. Opportunities for participation in research projects. Publications. Collectively seen as improved scholarship skills
8​	Woods, Attwell, Ross, Theron (2012) Text messages a learning tool for midwives Reference: [30]	South Africa	Midwives in clinical practice supported by Perinatal education programme staff	Short essential learning text messages with links to coursework sent to participants	Cost-effective and relevant clinical-content of messages was useful to those in both private and public sector, urban and rural setting	Participant enjoyment, improved their clinical practice, regular sharing and discussions about the messages with colleagues. Cost-effective learning opportunities, which can contribute to student teaching in clinical setting

#### Context

This review found that online faculty development initiatives, situated in low- and middle-income countries [26–33], are often offered in partnership with or through funding from US-based organisations, presented either formally or informally. The researchers acknowledge that a limitation of the study is that only articles published in English were included in this review. Formal initiatives included postgraduate degree programmes [31], compulsory institutional programmes for staff [26], or fellowship programmes [27–30]. Informal initiatives incorporated a journal club [32] or a once-off research study to investigate the rollout of an intervention for clinicians [33]. Volunteer health professions educators [27–30] or peer mentors [27–29, 32] within the programme itself were the primary facilitators in online faculty development [27–30]. Clinician researchers [33] and academic staff [26, 31, 32] facilitated online faculty development initiatives formally and informally. Participants in online faculty development programmes were academic staff or clinicians in various health profession disciplines [26–33].

#### Mechanism

Interventions (Table 1: Resources) within the reviewed studies made use of email discussions [26–28, 30, 32], online forum discussions [26, 32] and web-based modules [26, 29, 31]. The studies also reported face-to-face workshops and training opportunities used in a blended approach to faculty development [26, 29, 31]. However, most of the studies reported on interventions centred on the use of email discussion groups [26–28, 30, 32]. The approach utilised was predominantly topic-based discussions with a focus on either research or teaching skills development topics. The data presented within the reviewed articles strongly support the use of discursive practices between participants and facilitators as key resource mechanisms for online faculty development success.

#### Outcome

Online faculty development opportunities had both positive and negative outcomes related to engagement. These reported outcomes were identified by the researchers from descriptive post-implementation evaluations based on data collected during and after interventions by authors in the included studies. There was an increase in participation in a community of practice [26, 27, 30, 32], increased satisfaction [26, 32, 33] and the facilitation of networking [28–30]. In one study [31], adverse outcomes relating to engagement included difficulty in managing group dynamics and the lack of motivation of participants due to staff perceiving HPE programmes as a “bandwagon phenomenon” often linked to career progression requirements [31]. There was a greater level of reporting on professional development [26, 28–30, 32, 33] than career/capacity development [29], though both were outcomes of online faculty development opportunities. Where the design of a course facilitated learning in a safe, flexible and reflective learning environment [26, 28, 31], it also enabled the enhancement of the online faculty development opportunity [26–28, 31] and, in some cases, the design of new programmes [27–31, 33]. A focus, or lack thereof, on student-centred learning design resulted in positive [26–33] and negative outcomes [28, 31], respectively. The online faculty development opportunities were designed to deliver topical content. However, studies caution the need for awareness of online learnings' weaknesses, such as connectivity requirements and the lack of interaction created by the programmes' asynchronous nature [28, 31]. Online faculty development opportunities required facilitators who appropriately guided participants by facilitating communication and discussion [27–33]. These studies identified both the negative and positive outcomes of providing feedback or lack thereof [28, 31].

### Thematic outcomes

Using an inductive thematic analysis approach, the primary research team identified thirteen relevant themes within the CMO configuration data. Themes related to the Context [26, 27, 29, 30, 32, 33] were the geographical location, programme type, facilitator and participant roles and responsibilities. Themes such as discussion and collaboration highlight the engagement within the Mechanism [26–30, 32, 33] construct. In contrast, content delivery themes, self-directed learning, and evidence of competence suggested a focus on individual student activity. Finally, themes within the Outcomes [26–30, 32, 33] construct aligned clearly with the faculty development initiative's intended and perceived outcomes. These themes were engagement between facilitators and participants within a course, development in personal and professional domains, design of learning environments, and facilitator qualities related to course facilitation skills.

### Conjecture mapping

The final phase of the analysis consolidated the first phases with the foundational Conjecture Map and candidate theories used to set the review's objectives. The first author identified the overlap between the Conjecture Map components and the Community of Inquiry, aligning each theme with the relevant components and constructs (Table 2).

**Table 2:** Overlap of CMO, candidate theory and conjecture map

Community of Inquiry	Conjecture Map	Theme	CMO
Cognitive Presence	Embodiment	Geographical	Context
Teacher Presence	Embodiment	Programme type	Context
Teacher Presence	Embodiment	Facilitator	Context
Social Presence	Embodiment	Participant	Context
Cognitive, Social & Teacher Presence	Embodiment & Mediating Processes	Discussion	Mechanism
Social & Cognitive Presence	Embodiment & Mediating Processes	Collaboration	Mechanism
Cognitive & Teacher Presence	Embodiment & Mediating Processes	Self-directed learning	Mechanism
Teacher Presence	Embodiment & Mediating Processes	Content delivery	Mechanism
Cognitive & Teacher Presence	Embodiment & Mediating Processes	Evidence of competence	Mechanism
Social & Teacher Presence	Embodiment & Outcome	Engagement	Outcome
Cognitive Presence	Outcome	Development	Outcome
Cognitive, Social & Teacher Presence	Embodiment & Outcome	Design	Outcome
Teacher Presence	Embodiment	Facilitator Qualities	Outcome

**Fig. 3:**
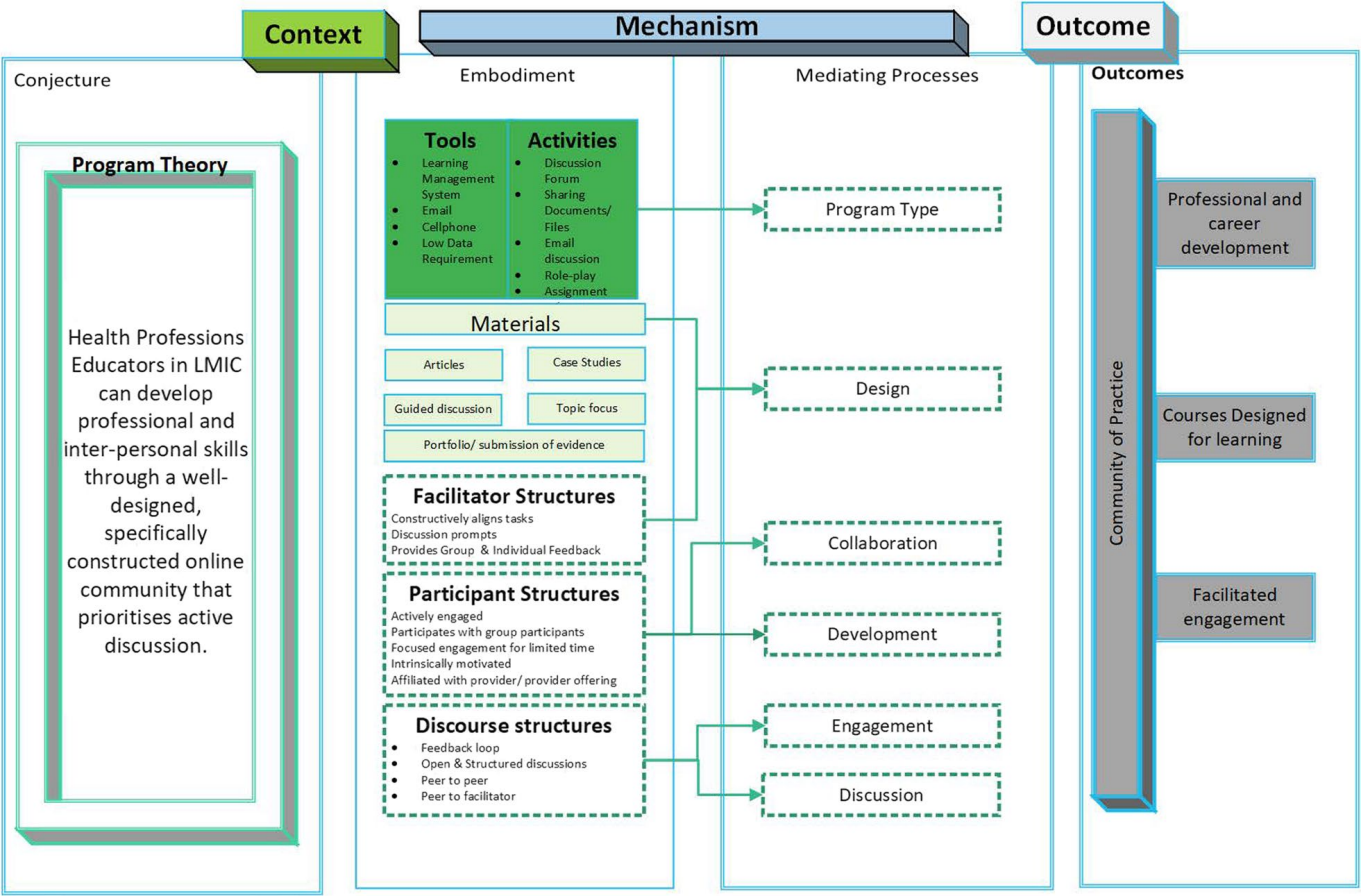
Conjecture map for online HPE faculty development

This alignment allowed the research team to refine and define the Conjecture for online health professions education faculty development in low- and middle-income countries. This educational design tool is now a potential research catalyst for faculty developers (Fig. 3).

## Discussion

This rapid realist review used a Conjecture Map developed with the Conversational Framework and Community of Inquiry as the theoretical basis for online health professions education faculty development courses' design considerations. The findings of the review partially supported selecting these candidate theories, specifically the Community of Inquiry, as underpinning asynchronous online learning. The programme theory was not reliant on the technical or design considerations of online faculty development for health professions educators, which would have supported the Conversational Framework as a core theory influencing course design. The Conversational Framework provides the parameters from which to assess the role of using feedback from the learning environment, the student and between students to adapt the design of the learning environment [10]. Further confirmation of the appropriate candidate theories emanated from three articles that reported a theoretical framework for evaluating their faculty development opportunity [27, 29, 31], two of which incorporated the Community of Inquiry into the design and evaluation of their faculty development opportunity [27, 31]. The third article applied the Kirkpatrick evaluation model to their study [29]. This outcome, paired with the CMO analysis, implies that the mechanism through which faculty development outcomes in low- and middle-income countries are achieved may be due to creating a community of educators, thus answering the “Why online faculty development courses in low- and middle-income countries work?”.

The existence or development of a community of educators facilitates collaborative learning within higher education [11]. In the context of faculty development, this review demonstrates the validity of interrogating the effectiveness of designing interventions through the lens of a community of practice [4]. This community should be focused on capacity development through collaborative learning and practice [29]. Furthermore, research has shown that faculty development should adopt an approach in which a community of practice is a critical outcome [34] in face-to-face and online initiatives [3].

Similar to earlier research identifying a positive correlation between cognitive presence and community [35], this review found that the positive developmental outcomes within well-designed, purpose-driven online courses consistently relied on the domains and categories that align with the Community of Inquiry and a Community of Practice. When an online course creates a mental discomfort secondary to contradictory information also known as "cognitive dissonance", the Community of Inquiry posits that cognitive dissonance is a stimuli or a triggering event for cognitive development [8]. In the process of theory building within this review, the authors found that the "triggering event" aligned with the Cognitive Presence in the Community of Inquiry is not specifically the existence of a sense of puzzlement or being lost in meaning [36], but rather the creation or existence of a Community of Practice. Aligning the themes—the Conjecture Map, the CMO configurations and the Community of Inquiry—highlights the recurrence of the teacher and cognitive presence constructs in these interventions.

This review identified six triggering events as critical in developing a community of practice in an online faculty development course for health professions educators in low- and middle-income countries. These are programme type, programme design, discussion, engagement, development, and collaboration. These events align with the Conjecture Map, predominantly within the embodiment of the course, outlining the salient design principles that include the tools, activities, materials, participant structures and discursive practices. Each of these either creates a "common ground for sharing knowledge", "a social structure for interactions", or creates and curates "specific knowledge shared, developed and maintained by the community [4]." Utilising the Community of Practice as a lens through which to analyse the design and theoretical conjectures for online health professions education courses is supported through the intended application of this theory [37] (Table 3).

**Table 3:** Alignment of triggering events with CMO

Article	Context		Mechanism		Outcome	
	Geographical consideration	Programme type	Discussion	Collaboration	Engagement	Development
Ahmed (2013) Tailoring online faculty development programmes: overcoming faculty resistance	√	√	√	√	√	√
Anshu, Sharma, Burdick & Singh (2010) Group Dynamics and Social Interaction in a South Asian Online Learning Forum for Faculty Development of Medical Teachers	√	√	√	√	√	√
Dongre, Chacko, Banu, Bhandary, Sahasrubudhe, Philip & Deshmukh (2010) Online Capacity-Building Program on "Analysis of Data" for Medical Educators in the South Asia Region: A Qualitative Exploration of our Experience	√	√	√	√	√	√
Frantz, Bezuidenhout, Burch, Mthembu, Rowe, Tan, Van Wyk & Van Heerden (2015) The impact of a faculty development programme for health professions educators in sub-Saharan Africa: an archival study	√	√	√	√	√	√
Ladhani, Chhatwal, Vyas, Iqbal, Tan & Diserens (2011) Online role-playing for faculty development	√	√	√	√	√	√
Naeem & Khan (2019) Stuck in the blend: Challenges faced by students enrolled in blended programs of Masters in Health Professions Education	√	√	√	√	–	–
Thakurdesai, Ghosh, Menon, Sahoo, Tripathi, Harshe & Andrade (2018) Electronic journal clubs for capacity building: A case study in psychiatry as a model for medical disciplines in developing countries	√	√	√	√	√	√
Woods, Attwell, Ross, Theron (2012) Text messages a learning tool for midwives	√	√	–	–	√	√

The authors answered the "what works, in what context, and why it works" within this review. In the light of these, the authors interrogated the data to identify "for whom" these initiatives work effectively. Considering the original intention of this review and efforts to ensure an approach to design and delivery of non-institutional online health professions education faculty development opportunities specifically for the low- and middle-income country context, there is a question of whether the foreign gaze [38] evident within the data is necessary for achieving the presented outcomes.

Literature and reviews have raised the notion of the foreign gaze in the context of Global Health [38]. Similarly, in the field of faculty development for health professions educators, the focus is primarily on developed countries [6], written by and for, that is, the pose and gaze in relation to international audiences [38]. The question that the finding of this review raises is whether this can be attributed to publications that are more likely to emanate from a foreign gaze perspective, assuming what works in low- and middle-income countries. The reviewed articles were primarily US-based in origin, ownership of the intervention or authorship profile, thus written with a foreign pose and with a foreign gaze to international audiences. While the authors concede that the triggering event of programme type could be an internationally funded and resourced intervention, the question is whether the foreign gaze is a necessary catalyst for developing the resultant online community in a low- and middle-income context. Faculty development initiatives in Africa have incorporated communities of practice as a capacity development opportunity [39]. However, in the study presenting this finding, US funding was relied on in a "partnership", calling into question cultural alignment with the recipients' needs at the inception of the programme [2]. The answer to the question relating to "for whom?” online health professions education faculty development courses work indicates educators and clinical educators as the recipients. This review cannot unequivocally state that this is true for facilitators from low- and middle-income countries, as the facilitators in all studies were a combination of both local and foreign.

As an outcome of this rapid realist review, the programme theory generated is that "health professions educators in low- and middle-income countries (context) can develop professional and inter-personal skills (outcome) through a well-designed, specifically constructed online community that prioritises active discussion (mechanism)".

### Strengths, limitations and future research recommendations

This review's design followed an iterative process with multiple blinded reviewers across a primary and secondary research team. Each component of the structured approach [21, 22] adopted by the research teams was documented and reviewed throughout the analysis phase. The design and process employed demonstrate the rigour applied to the review, limiting the bias and potential gaps that the authors may have encountered. In addition, initiating this review by utilising a Conjecture Map to integrate the educational and theoretical focus as the subject under investigation further strengthens the study. The limitation of this study was the absence of an initial consultative process with the health professions education community members to identify candidate theories, as is dictated through the original realist review methodology [22]. As a mitigation strategy for this limitation, the authors sought input on the study protocol from health professions educators at two higher education institutions in South Africa. The authors recognise the limitation that English papers, those identified using the search terms focused on Africa and not inclusive of specific health professions could limit the number of citations that may have met the inclusion criteria. However, the university library in South Africa that was accessed for the review had institutional access to most internationally recognised popular databases. The reviewed articles' quality and theoretical contribution are not uncommon in online education literature and require higher quality standards in the research conducted [1–4, 20]. The use of a Conjecture Map supports an approach to strengthening this and future research on the theoretically grounded design of online health professions education faculty development courses [40].

Future research should include more comprehensive consultation across health professions educators to investigate the reliability and feasibility of these findings about the design and delivery of online health professions education faculty development in low- and middle-income countries.

## Conclusion

This rapid realist review aimed to answer how online health professions education faculty development courses work or do not work in low- and middle-income countries. The approach to executing this review highlights the possibilities of integrating educational design research tools for the development of candidate theories and the realist review approach. This review's findings demonstrate the community's role as the dominant mechanism through which these interventions work, based on a design that incorporates six triggering events. The study aligns these events with the three categories of the Community of Inquiry, a theory applicable to the design of online learning environments. Health professions educators in low- and middle-income countries can develop professional and interpersonal skills through a theory-based purposeful online community that prioritises active discussion. Finally, this rapid realist review highlights deficiencies in the quantity publications and the influence of a foreign gaze on the evidence available within this field. As part of a larger research project, this study supports our final recommendation that further research is required to contextualise these findings through a consultative process to develop a model for designing and delivering online health professions education faculty development courses within a low- and middle-income setting.

## Availability of supporting data

Relevant data and materials have been provided as Additional Files 1 and 2, referenced in the manuscript.

## Supplementary Information

**Additional file 1:** Search Process.

**Additional file 2:** Data Extraction Tool.

## Abbreviations

HPE: Health professions education
CoP: Community of Practice
CMO: Context, mechanism, outcome
Online HPE-FD: Online Health Professions Education Faculty Development
PICO: Population, intervention, context and outcome
LMIC: Low- and middle-income countries
US: United States of America
