# Supplementary material for: Online faculty development in low- and middle-income countries for health professions educators: a rapid realist review
Source: Hum Resour Health. 2022 Jan 29;20:12. doi: 10.1186/s12960-022-00711-6 (PMC8799968; doi:10.1186/s12960-022-00711-6)
Supplement: Supplementary file 1 — Additional file 1: Search Process. [file 12960_2022_711_MOESM1_ESM.pdf]

	Total hits	Automatic duplicates removed	Hits after automatic deduplication	Duplicates within search	Unique hits	Duplicates across searches	Total duplicates
**Search 1**	310	53	257	55	202	75	183
**Search 2**	138	34	104	22	82	0	56
**Search 3**	1388	500	888	43	845	24	567
	**1836**	**587**	**1249**	**120**	**1129**	**99**	**806**

Final Unique	Selected	Did not meet criteria	Full texts available	Full texts selected first review	Ancestor search	Duplicates in ancestor search	Ancestor search abstracts	Ancestor search selected
127	14							
82	16							
821	20							
**1030**	**50**	**980**	**49**	**9**	**10**	**2**	**8**	**0**

Full texts selected for review
9
