# Supplementary material for: Online faculty development in low- and middle-income countries for health professions educators: a rapid realist review
Source: Hum Resour Health. 2022 Jan 29;20:12. doi: 10.1186/s12960-022-00711-6 (PMC8799968; doi:10.1186/s12960-022-00711-6)
Supplement: Supplementary file 2 — Additional file 2: Data Extraction Tool. [file 12960_2022_711_MOESM2_ESM.pdf]

Reviewer	Author	Title
R1	Ahmed	Tailoring online faculty development programmes: overcoming faculty resistance
R4		
R5		

R1	Anshu, Sharma, Burdick & Singh	Group Dynamics and Social Interaction in a South Asian Online Learning Forum for Faculty Development of Medical Teachers
R4		

R5		
R1	Dongre, Chacko, Banu, Bhandary, Sahasrubudhe, Philip & Deshmukh	On-line Capacity-Building Program on “Analysis of Data” for Medical Educators in the South Asia Region: A Qualitative Exploration of our Experience

R4		
R5		

R1	Frantz, Bezuidenhout, Burch, Mthembu, Rowe, Tan, Van Wyk & Van Heerden	The impact of a faculty development programme for health professions educators in sub-Saharan Africa: an archival study
R4		
R5		

R1	Ladhani, Chhatwal, Vyas, Iqbal, Tan & Diserens	Online role-playing for faculty development

R4		
R5		

R1	Naeem & Khan	Stuck in the blend: Challenges faced by students enrolled in blended programs of Masters in Health Professions Education

R4		
R5		

R1	Thakurdesai, Ghosh, Menon, Sahoo, Tripathi, Harshe & Andrade	Electronic journal clubs for capacity building: A case study in psychiatry as a model for medical disciplines in developing countries

R4		
R5		

R1	Vovides, Chale, Gadhula, Kebaestse, Nigussie, Suleman, Tibyampansha, Ibrahim, Ntabaye, Frehywot & Nkomazana	A Systems Approach to Implementation of eLearning in Medical Education: Five MEPI Schools' Journeys
R4		
R5		

R1	Woods, Attwell, Ross, Theron	Text messages a a learning tool for midwives
R4		

R5		

Year of publication	Country where research was completed
2013	Egypt
2013	Eqypt
2013; pg 535	Egypt, pg 535

2010	India
2010	India (p2)

2010 pg 1	India pg 1
2010	India, Nepal, Malaysia p4

2010	India (p2)
2010 pg 1	India Nepal Malaysia pg 1

2015	11 African countries, South Africa, Uganda, Sudan, Zambia,Zimbabwe, Malawi, Mozambique, Tanzania, Nigeria, Madagascar, Botswana p4
2015	South Africa (p1)
2015 pg 1	South Africa pg 1

2011	four different countries (two from India, three from Pakistan, and one each from Malaysia and the USAp33

2011	Globally
2011; pg 31	Pakistan India Malaysia USA pg 31

2019	Pakistan

2019	Pakistan (p2)
2019 pg 929;	Pakistan pg 929

2018	India

2018	India (p1)
2018 pg 93	India pg 93

2014	Tanzania, Ethiopia, South Africa, Zimbabwe, Botswana
2014	Ethiopia, Tanzania, Botswana, South Africa and Zimbabwe
2014; pg s102	Ethiopia S102 Botswana S103

2012	South Africa
2012	South Africa (p1)

2012, pg 1	South Africa pg 1

Discipline	Facilitator recruitment
Ophthalmology and Vascular Surgery	expert from outside the institute
Vascular Surgery	from outside the institute was added
Ophthalmology and Vascular Surgery. Pg 535	An expert from outside the institute was added pg 535

from lecturers with one to two years experience in teaching to professors with over two decades of experience	Batch 1 fellows
No specific discipline as it comprises several health professions within the CMCL-FAIMER fellowship program<br><br>Regional Institute at the Christian Medical College, Ludhiana (CMCL) (p2)	The fellows then volunteer to moderate sessions on topics of their interest (p3)

Medical pg 1	fellows then volunteer to moderate sessions on topics of their interest. Pg 3
medical educators from different disciplines of Medicine p4	Four fellows (2 each from year 2008 and year 2009) were the moderators under the supervision of two PSG-FRI faculty members P4

No specific discipline as it comprises several health professions within the PSG-FAIMER<br><br>PSG-FAIMER Regional Institute (PSG-FRI), Coimbatore,<br>India (p2)	a team of four discussion moderators (p3)<br><br>second-year fellows who had already completed their CIPs the previous year and were also professionally trained in the field of qualitative and quantitative data analysis (p3)
different disciplines of Medicine pg 4	Four fellows (2 each from year 2008 and year 2009) were the moderators under the supervision of two PSG-FRI faculty members.pg 4

Medicine, Nursing, Dentistry, Allied Health, Education specialist (Health Professions Educators) p4	African health professions educators p3
No specific discipline as it comprises	community of African health
Health professions education pg 1	

None Specified	discussions are made more meaningful by assigning a pre-selected topic to a group of between four and six fellows and a faculty member for leading and moderating the discussion each month p32 The moderators, six FAIMER fellows and a faculty member p33

No specific discipline as it comprises several heatlh professions<br><br>Foundation for Advancement<br>of International Medical Education<br>& Research (FAIMER)	The moderators, six FAIMER□ fellows and a faculty member, were located in four different countries (two from India, three from Pakistan, and one each from Malaysia and the USA) (p3)
health professions education pg 32	The moderators, six FAIMER?? fellows and a faculty member six FAIMER?? fellows and a faculty member, were located in four different countries (two from India, three from Pakistan, and one each from Malaysia and the USA) pg 33

Health Professions Education p929	MHPE program facilitators

Masters of Health Professions Education (p1)	Facilitators from the MHPE programme
Health Professions Education pg 929	Evaluation: four facilitators were included from three universities from three different cities of Pakistan. pg 930<br><br>“We do not have very good ratio of faculty to students, and mostly faculty is visiting.” p931

Psychiatry	Task Force of the Indian Psychiatric Society on Psychopharmacology, along with the Task Force of the Indian Psychiatric Society on Workshops and Training, and with the support of the Department of Psychopharmacology at the National Institute of Mental Health and Neurosciences (NIMHANS), Bengaluru, India (Sathyanarayana Rao and Andrade, 2015a,b), it is presently operated and moderated only by the Department of Psychopharmacology, NIMHANS. P94

predominantly comprised<br>of psychiatrists (p2)	The senior members of the group,<br>usually the moderator,<br>from the Department of<br>Psychopharmacology, NIMHANS,<br>periodically<br>initiate and moderate discussions<br>(described as 'Activities'); sometimes,<br>discussions are also initiated by<br>junior members. (p3)
Psychiatry pg 93	The senior members of the group,<br>usually the moderator, from the<br>Department of Psychopharmacology,<br>NIMHANS, pg 95

University faculty S102 medical school faculty S103, Pharmacy Faculty S104	participants in the Medical Education Partnership Initiative (MEPI) eLearning Technical Working Group S102
Medicine and pharmacy	Medical Education Partnership Initiative (MEPI) eLearning Technical Working Group (p1)
Medicine s103 Pharmacy (UKZN)	

Midwifery	Perinatal Education Programme (PEP) Staff P101
Midwifery (p1)	Perinatal Education Programme (PEP) (p2)

midwives pg 1

Recipient recruitment	Faciliatator qualities
76 faculty<br>members were given the choice of<br>using web-based modules,<br>and e-mail-based discussion forums<br>to promote selfdirected<br>learning.	expert from outside the institute
to-face training sessions	Not described
76 faculty members were given the<br>choice pg 535	

Online session with Batch 2 as primary participants.	Batch 1 fellows provide mentoring support.
Regional Institute at the Christian Medical College, Ludhiana (CMCL) (p2)	Not described

15 individuals were selected into the fellowship from 12 medical schools in five different states of India. Pg 3	The key to successful moderation was to guide the group towards various kinds of inputs relevant to the topic under discussion, rather than for faculty or moderators to directly provide material. Pg 7
30 fellows (14 from year 2008 and 16 from year 2009) p4 PSG-FAIMER Regional Institute (PSG-FRI), Coimbatore,<br>India during October 2009 p2	second-year fellows who had already completed their CIPs the previous year and were also professionally trained in the field of qualitative and quantitative data<br>analysis p3

PSG-FAIMER Regional Institute (PSG-FRI), Coimbatore,<br>India (p2)	second-year fellows who had already completed their CIPs the previous year and were also professionally trained in the field of qualitative and quantitative data analysis (p3)
30 fellows (14 from year 2008 and 16 from year 2009) from different states of India, Nepal and Malaysia. Pg 4	The second week's moderation was led by second-year fellows who had already completed their CIPs the previous year and were also professionally trained in the field of qualitative and quantitative data analysis. Pg 3

Each year FAIMER awards 16–18 fellowships to sub-Saharan African health professions educators p2	health professions educators who teach, practise and conduct HPE-related research in a wide range of sub-Saharan African countries. P3
health professions educators (p2)	teach, practise and conduct HPE-
Each year FAIMER awards 16–18 fellowships to sub-Saharan African health professions educators pg 2<br><br>Evaluation: 64 health professions educators representing 11 African countries	African health professions educators who teach, practise and conduct HPE-related research in a wide range of sub-Saharan African countries pg 3.

During the distance-learning sessions, fellows participate in a series of online discussions on a mailing list server in which all current and former fellows and faculty members are involved p32 The list server group consisted of approximately 120 fellows and faculty members, representing around 30 countries over four continents p33	FAIMER fellows and a faculty member p33

The list<br>server group consisted of<br>approximately<br>120 fellows and faculty<br>members, representing around 30<br>countries over four continents (p3)	Not described
The list server group consisted of<br>approx-imately 120 fellows and<br>faculty members representing<br>around 30 countries over four<br>continents pg 33	

Eighteen students from three universities from three different cities of Pakistan p930	MHPE program facilitators

Students from the MHPE programme	Not described
Evaluation : Eighteen students were included from three universities from three different cities of Pakistan. pg 930<br><br>Fourteen students joined the program because of being 'in trend'. Other reasons quoted by students included career up gradation, personal interest, feasibility as well as being recommended by others. p931	Varying teaching styles with conflicting views pg 931 The Facilitators play a myriad of roles in blended programs, from instructor to mentor and by providing timely feedback, and being approachable, they can facilitate quality learning. Pg 933

No strictures are placed on applications for and approval of eJCIndia memberships P94	senior members of the group, usually the moderator, from the Department of Psychopharmacology, NIMHANS P95

All takes place on the JCIndia platform	Not described
The membership is predominantly comprised of psychiatrists. Pg 94 No strictures are placed on applications for and approval of eJCIndia memberships. Pg 94	members are welcome to ask questions and share knowledge regardless of their level of seniority pg 94 knowledge pooling by members who have expertise in di?erent ?elds of psychiatry and psychiatric research.pg95

University faculty S102 medical school faculty S103, Pharmacy Faculty S104	None
eJCIndia provides an e-platform for discussing and imparting training in academic and research-related issues (p2)	Not described, but part of the MEPI eLearning Techincal Working Group
Ethiopia: Doctors and other health	

cell phone numbers of more than 2 500 midwives were provided by the Sister Lilian Centre, which produces a quarterly nursing magazine, Sensitive Midwifery, and manages annual, popular and well-attended refresher courses for midwives P101	Perinatal Education Programme (PEP) Staff P101
The cell phone numbers of more than 2 500 midwives were provided by the Sister Lilian Centre (p2)	Not described

cell phone numbers of more than 2 500 midwives were provided by the Sister Lilian Centre, pg 2 ; 50 recipients were randomly identified: 25 in the public and private sectors, respectively. These midwives were contacted by an independent consultant,	

Recipient Professional role/Position	Faculty development initiative
Faculty members	case designed according to the objectives of the course, which was e-mailed to all participants, with an in-built assignment to complete and return by e-mail. Each submission was assessed individually. Based on the gaps in their learning, educational objectives were defined and an individual assignment was designed for each faculty member to address his or her individual objectives. Online discussions on one or two themes were facilitated to clarify doubts. This was followed by a group assignment. An expert from outside the institute was added to the mailing list and asked to look at the assignment and to provide feedback over a span of 3 weeks, during which learning was asynchronous at the faculty member's own pace. This was followed by a 3-hour face-to-face training encounter. Certificates of completion were given at the end of the course.
Department of origin, though	to-face training sessions
Ophthalmology and Vascular Surgery. Pg 535	choice of using web-based mod-ules, and e-mail-based discussion forums to promote self-directed learning. Pg 535

15 individuals were selected into the fellowship from 12 medical schools in five different states of India. Their ages when<br>selected were between 27 and 49 years, with a median of 33 years. Their academic ranks ranged from lecturers with one to two<br>years experience in teaching to professors with over two decades of experience. (3)	‘Mentoring and Learning Web
Not described further than the fellowship	Fellows select the topics<br>for discussion in the monthly ML Web sessions by multi-voting. Multi-voting is a standard tool to arrange and order a list of ideas<br>by importance. It is a group effort where every member of the group is allowed to give a number ranking the importance to each<br>suggested topic. Those topics receiving highest rankings from the group get further attention. The fellows then volunteer to<br>moderate sessions on topics of their interest. (p3)

medical teachers pg 2 academic ranks ranged from lecturers with one to two years experience in teaching to professors with over two decades of experience. Pg 3	one month's ML Web session, on the topic 'Web-based Learning'. Pg 3
medical educators from different disciplines of Medicine having more than three years of teaching experience.	to ensure timely technical help in analysis of CIP-generated data, an M-L Web discussion on the topic 'Analysis of Data' was undertaken on the listserv p2

Not described further than the fellowship	Web) (p2)<br><br>It is an on-line component of the faculty leadership development program by “The FAIMER Institute”, which begins with a residential session at the regional center followed by an 11-month, on-line M-L Web discussion through listserv on topics of stated interest by fellows. This series of month-long intersession activity is moderated by fellows and regional faculty members (p2)<br><br>an M-L Web discussion on the topic ‘Analysis of Data’ was undertaken on the listserv of the PSG-FAIMER Regional Institute (PSG-FRI), Coimbatore, India (p2)<br><br>The first half of the month was devoted to capacity-building on quantitative methods and the second half to qualitative methods. During each of these halves of the month, the first week was devoted to giving
medical educators from different disciplines of Medicine pg 4	on-line capacity-building in quantitative data analysis pg 4

sub-Saharan African health professions educators p2	SAFRI faculty development programme focuses on developing African health professions educators as leaders, teachers, scholars and advocates for change at multiple levels, including the individual, school, university and the health professions p2
health professions educators (p2)	This two-year programme, a
a wide range of health professions educators pg 1	The SAFRI faculty development programme focuses on developing African health professions educators as leaders, teachers, scholars and advocates for change at multiple levels, including the individual, school, university and the health professions. Pg 2

health professions educators p32	An online role-play scenario-based discussion was planned p33 On behalf of ‘the dean’ an e-mail containing the aforementioned scenario was sent to the the groups’ list server inviting all FAIMER fellows to build a case for CBME. In addition, two articles related to CBME were sent as resource materials for everyone. The scenario and the fictitious names encouraged the participants who spontaneously started using virtual roles akin to their beliefs or thoughts, thus creating characters with a certain point of view. For example, Dr Up-to-Date’s comments and ideas were in favour, whereas another fellow who replied as Dr Real Healthcare challenged the effectiveness of CBME. Another participant on the list server was Dr Atom Technetium who raised logistical concerns about implementing the concept of CBME in medical schools. The momentum of such a rich discussion was maintained throughout the month as each member of the moderating

health professions'<br>educators (p2)	An<br>online role-play scenario-based<br>discussion was planned (p3)
health professions' educators pg 32	•To stimulate active discussion on the topic and present evidence for CBME implementation;<br>•To enhance the participants' understanding pertaining to CBME;<br>•To engage the group members as online distance learners.pg 32

None	rotation model, Masters of Health Professions Education (MHPE) in Pakistan p929

Eighteen students (p2)	Blended programs of Masters of Health<br>Professions Education (MHPE) in Pakistan (p1)
Health professions varying disciplines Pg 929	Blended programs of Masters of Health Professions Education (MHPE) pg 929

postgraduate students, some are recently graduated psychiatrists, and a<br>sizeable number are psychiatrists who are academic faculty in teaching institutions. eJCIndia has only a small representation from among psychiatrists who are solely in practice P95	an e-platform for discussing and imparting training in academic and research-related issues. It aims to develop member skills in critically reading a research<br>paper, to clarify issues related to research methodology (ranging from study design to choice of appropriate research instruments for use in specific contexts to methods of statistical analysis of data) and to improve member skills in scientific writing. The most important mission of eJCIndia is to train trainees and their trainers; that is, to strengthen academic and research capacities in departments of psychiatry across the country so that future generations are benefited. P94

postgraduate students, some are recently graduated psychiatrists, and a<br>sizeable number are psychiatrists who are academic faculty in teaching institutions. eJCIndia has only a small representation from among psychiatrists who are solely in practice. (p3)	As with traditional JCs and eJCs, eJCIndia provides an e-platform<br>for discussing and imparting training in academic and research-related issues. It aims to develop member skills in critically reading a research paper, to clarify issues related to research methodology (ranging from study design to choice of appropriate research instruments for use in specific contexts to methods of statistical analysis of data) and to improve member skills in scientific writing (p2)
Most of the members are postgraduate students, some are recently graduated psychiatrists, and a sizeable number are psychiatrists who are academic faculty in teaching institutions. eJCIndia has only a small representation from among psychiatrists who are solely in practice. pg 94-95	elecronic journal club pg 92

Faculty members	An eLearning steering committee was established, composed of faculty from different disciplines and ICT personnel, to enhance two-way communication concerning the use and benefits of these technologies. Training workshops on Moodle, instructional design, and use of the E-library platform eGranary were also held, and additional ICT support personnel were hired S102-103... Regular LCMS+ training and refreshers have been offered to the faculty and students S103 Ongoing training and support S103 training for the academic staff in Moodle to be offered centrally S104
four African medical schools and one school of pharmacy (p1)	of eLearning implementation, the core components to be addressed are institutional support, faculty engagement, student engagement, technical expertise, and infrastructure and support systems (p1)<br><br>There are many posts to eJCIndia that require no discussion. Examples of such posts include links to important, recently-published articles; links to important websites (such as a website that teaches visitors how to read magnetic resonance imaging scans, or websites for online data analysis); a link to a tutorial on how to efficiently use PubMed to search the medical research literature; or even merely an article on how to do a Boolean search for online resource materials. The purpose of such posts is to improve
Ethiopia: faculty s102	eLearning in Medical Education s102

midwives in the South African public and private sectors P101	Twenty-six short, essential learning messages were selected from the Maternal Care course book of the PEP. Each message, limited to 160 characters, was sent via text message to the midwives every Tuesday over a 6-month period. Each message ended with a link to the Sister Lilian website, which in turn linked to the publisher's website, where the corresponding Maternal Care chapter could be read p101
midwives (p2)	A recent project has brought essential healthcare lessons from the Maternal Care book of the Perinatal Education Programme (PEP) to thousands of midwives through the medium of short message service (SMS) text messaging, using basic cell phones. (p2)

midwives pg2	essential healthcare lessons from the Maternal Care book of the Perinatal Education Programme (PEP) pg 2

Theoretical/Conceptual Model Used	Method of delivery/Tools used
None	Email, Discussion group, Group assignments, Individual assignments, F2F training
Not described	and e-mail-based discussion forums
more tailored solutions to accommo-date faculty needs. Pg 535	web-based mod-ules, and e-mail-based discussion forums pg 535

modification of the Community of Inquiry model (3)	Email (ListServe)
Not described	CMCL-FAIMER listserv during the ML Web sessions. (p3)

Social presence', or the degree to which participants in online communication feel affectively connected to one another, is important for student satisfaction and success in online courses Pg 2	online session, called Mentoring and Learning Web' (ML Web) lasts 11 months pg 2-3
adult learning principles p3	an on-line survey using a structured questionnaire covering quantitative data analysis and five open ended questions relating to qualitative data analysis issues was mailed on the listserv, shared listserv discussion p3

adult learning principles (p3)	an M-L Web discussion<br>on the topic ‘Analysis of Data’ was undertaken on the listserv of the PSG-FAIMER Regional Institute (PSG-FRI), Coimbatore,<br>India (p2)
adult learning principles pg 3	on-line Mentoring and Learning Web discussion through listserv Pg 4

	(1) three residential sessions of five to nine days each, (2) a distance learning component and (3) the design and completion of an education innovation project which focuses on improving HPE in the home institution of each fellow
Not described	which includes (1) three residential
ducation and leadership faculty development programme, which aims “to strengthen med-ical education and to build a sustainable discipline of med-ical education in developing countries” pg 2	(1) three residential ses-sions of five to nine days each, (2) a distance learning component and (3) the design and completion of an education innovation project which focuses on improv-ing HPE in the home institution of each fellow pg 2

Role-playing p32	e-mail containing the aforementioned scenario was sent to thethe groups' list server p33

Adult learning (p4)<br><br>Role-playing (p1)	online role-play scenario-based discussion was planned (p3)<br><br>On behalf of ‘the dean’ an e-mail containing the aforementioned scenario was sent to the the groups’ list server inviting all FAIMER fellows to build a case for CBME. In addition, two articles related to CBME were sent as resource materials for everyone. The scenario and the fictitious names encouraged the participants who spontaneously started using virtual roles akin to their beliefs or thoughts, thus creating characters with a certain point of view. For example, Dr Up-to-Date’s comments and ideas were in favour, whereas another fellow who replied as Dr Real Healthcare challenged the effectiveness of CBME. Another participant on the list server was Dr Atom Technetium who raised logistical concerns about implementing the concept of CBME in medical
principles of adult learning, online learning and role-playing, which are inter-related and, when tied up together, increase the chances of effective outcomes pg 34	online role-play scenario-based discussion pg 33

constructivist-collaborative approach p930	traditional component of face to face (f2f) sessions, alternating with distance learning phase of two to three months p929

constructivist-collaborative<br>approach (p2)	Blended programs of Masters of<br>Health<br>Professions Education (MHPE) in<br>Pakistan have<br>increased from one in 2009 to eight<br>in 2018.1 These<br>programs are primarily designed on<br>rotation model,<br>including traditional component of<br>face to face (f2f)<br>sessions, alternating with distance<br>learning phase<br>of two to three months.2 (p1)
Adopting a constructivist-collabora-<br>tive approach, these MHPE programs<br>demand a sophisticated blend<br>between the needs of all stake-<br>holders (students, facilitators and<br>institutions) in order to maintain<br>interactivity and dynamicity they<br>offer. Pg 930	These programs are primarily<br>designed on rotation model,<br>including traditional component of<br>face to face (f2f) sessions, alternating<br>with distance learning phase of two<br>to three months.pg 929

None	closed email community

Not described	Not described
an internet-based learning platform that would help in devel-oping academic and research competence in India in the ?eld of mental health and the allied neurosciences. Pg 93	an e-platform for discussing and imparting training in academic and research-related issues. Pg 94 About 1–3 activities are conducted each week.pg 95

	Workshops, Training
Not described	eLearning (p2)
Ethiopia:To create access to self-	Ethiopia: Training workshops s102

	text message
Not described	Twenty-six short, essential learning messages were selected from the Maternal Care course book of the PEP. Each message, limited to 160 characters, was sent via text message to the midwives every Tuesday over a 6-month period. Each message ended with a link to the Sister Lilian website, which in turn linked to the publisher's website, where the corresponding Maternal Care chapter could be read.8 Therefore, midwives with internet access could retrieve additional information relating to the core message. The cost of sending the text messages was reduced by purchasing large bundles of messages; during the study period, 70 000 text messages were sent at a cost of 34 cents per message. (p2)

The use of cell phones to improve access to continuing education for midwives may help to address the need for ongoing training, especially in rural primary care facilities, that cannot be met by expensive, traditional methods of centralised, tutor-based training p1	Twenty-six short, essential learning messages were selected from the Maternal Care course book of the PEP. Each message, limited to 160 characters, was sent via text message to the midwives every Tuesday over a 6-month period. Each message ended with a link to the Sister Lilian website, which in turn linked to the publisher’s website, where the corresponding Maternal Care chapter could be read.8 Therefore, midwives with internet access could retrieve additional information relating to the core message. pg 2

Outcome	Recommendations
increase<br>in participation in the faculty development programme<br>from 8.3% (n = 6) to 42.1% (n = 32). Faculty member satisfaction<br>was measured using a pre-validated questionnaire.<br>The mean satisfaction index increased from 32.1 to<br>71.4 on a 1–100 satisfaction index. Faculty members considered<br>a mean percentage of 74.4% of their time spent<br>during training as useful as opposed to an initial 31.0%<br>when using traditional training methods. Faculty members<br>valued the training for being 'customer oriented' and<br>recognised that it 'respected what they needed more than<br>what the medical education department wanted to<br>deliver'. (p535) faculty members benefited from<br>and were satisfied with the training when it was tailored<br>to respect their privacy, their needs and their time	Giving them a safe learning environment<br>and an opportunity to study at their own convenient<br>time and place was more acceptable than being<br>coerced into a mandatory training programme.<br>Also, the opinion of an expert from outside the school is<br>a valuable tool and helps to cut down the resistance.
there was an increase	demonstrate that faculty members
an increase in participation in the faculty development programme from 8.3% (n = 6) to 42.1% (n = 32). The mean satisfaction index increased from 32.1 to 71.4 on a 1–100 satisfaction index. Pg 535	Faculty members bene?ted from and were satis?ed with the training when it was tailored to respect their privacy, their needs and their time. Pg 535

The 131 social emails were analyzed for content. Seventy-five (57.2%) were affective in nature. Most of these affirmed a fellow's belief or expressed an opinion on the ongoing discussion.(4) Cohesive emails (31, 23.6%) included posts by previous months' moderators thanking the group for their involvement, words of encouragement to other participants on their academic and personal successes, and positive remarks to otherwise quiet learners for their participation to get them involved in the discussion. (5) Twenty-five (19%) emails were interactive in nature and included light banter and playful exchanges between learners (5)	Moderators should not dissuade learners from bonding online. Moderators need to know when to intervene, how to provoke learners into responding and when to diffuse tensions. Social messages help moderators understand the needs of learners and then suitably alter the course content to meet their varying needs and interests. We believe that social interactivity helps in sustaining the group. It enhances the quality of discussions and the interest of the participants. Moderators must accept social interactions as inevitable and use them to enhance the quality of their courses.
found that the quality of participation was uneven and learners who were not familiar with the medium initially sent only social responses. This social interaction was not discouraged as it meant even the shy learners were reading the emails and gaining information. Eventually, the peer pressure to perform well ensured that all fellows read relevant literature and responded to the discussion. The average of almost 88 mails a week exchanged between learners is testimony to the ample, to and fro exchanges between them.<br><br>During the course of our online discussions, we found that affect was expressed in a number of ways, including through the use of emoticons, humor, and self-disclosure. Humorous banter, teasing, and joking allowed decreased friction and differences between	organizational and moderators set up the agenda for the discussion and manage interactions. They also have a social role of creating a friendly learning environment and an intellectual role of enhancing learning by asking pertinent questions and clarifying points. The main jelling factor is the social interactions between the people within the group. (p7)<br><br>We believe that social emails are inevitable on an online discussion forum. However, the art of effective moderation must be learnt so that the online learning activity can be streamlined into discussing relevant issues and does not become an inconsequential chat group.<br>Moderators should not dissuade learners from bonding online. Moderators need to know when to intervene, how to provoke learners into responding and when to

Social emails constituted one-third of the total emails. Another one-quarter of the emails dealt with sharing of resources and teacher presence, while cognitive emails comprised 36.2% of the total. More than half of the social emails were affective, while a little less than one-third were cohesive. pg 2	Moderators must accept social interactions as inevitable and use them to enhance the quality of their courses. Pg 8
among fellows who attempted competency-based exercises, only 4 (22.2%) had competency to test normality assumptions, 11 (52.4%) could do Likert scale analysis and 12 (60%) could apply appropriate tests of significance, need for discussion on such topics as: methods in qualitative research; poor attitude/faith in qualitative research; apprehension about its subjective nature; and lack of experience in handling such datasets and the reporting of qualitative research. Of the fellows, 19 had been using qualitative methods in their work setting, mainly, in the form of responses to open-ended questions. Sixteen fellows reported not receiving any formal training in these methods. Related to this, 12 fellows were not comfortable in the reporting of qualitative data. Barriers in this regard included: time-consuming nature of analysis; subjective nature of analysis; and perceived 'superior' nature of quantitative research.p4 ...the M-L Web brought together learners who	On-line learning is a flexible and convenient method of faculty capacity-building and overcomes limitations of geographical isolation p7

together learners who were geographically scattered and listserv discussion remained focused on the learning needs of the fellows. The M-L Web discussion offered learning on such topics as 'basic concepts in statistics' (normal distribution, variables), tests of significance, Likert scale analysis, bivariate correlation, and simple regression analysis and content analysis of qualitative data. There was discussion on perceived barriers to qualitative research methods such as its time-consuming nature, subjectivity and the perceived 'superior' nature of quantitative research. This timely discussion and feedback on the topic of data analysis is expected to benefit first-year fellows who are currently working on their CIPs, offering support in study design, implementation and analysis plans and production of technically robust research work. The exploration of e-mail contents	At the same time, potential weaknesses of on-line learning should be kept in mind, such as its impersonal approach, lack of spontaneous response compared to classes, fear of getting overloaded with information and links, need for special equipment and skills and access to internet services (p6)<br><br>steps such as identifying the learning needs for an on-line M-L Web discussion, addressing the immediate needs of the learners and a flexible reflective learning environment on the M-L Web facilitated collective learning outcomes of the fellows on the topic of data analysis. (p7)
identified the types of learning needs on the topic 'Analysis of Data' to be addressed for faculty development in the field of education research. This need-based M-L Web discussion could then facilitate collective learning on such topics as 'basic concepts in statistics', tests of significance, Likert scale analysis, bivariate correlation, and simple regression analysis and content analysis of qualitative data. pg 2	Identifying the learning needs for an on-line M-L Web discussion, addressing the immediate needs of the learners and a flexible reflective learning environment on the M-L Web facilitated collective learning outcomes pg 7

impact of the programme. These were grouped as: (1) belonging to a community of practice, (2) personal development, (3) professional development, (4) use of tools and strategies for project management and/or advancement, and (5) capacity development p4	programmes geared at health professions educators must be cogniscent of the local context in which participants work, teach and pursue the scholarship of HPE. Furthermore, the diversity demonstrated in the SAFRI programme demands a broad, context-sensitive knowledge of HPE as well as excellent teaching and research skills to run such programmes p8
The participants indicated that the	programmes geared at
Five key themes about the impact of the SAFRI programme were identified: (1) belonging to a community of practice, (2) personal development, (3) professional development, (4) capacity development, and (5) tools/strategies for project management and/or advancement. pg 1	programmes geared at health professions educators must be cogniscent of the local context in which participants work, teach and pur-sue the scholarship of HPE. Furthermore, the diversity demonstrated in the SAFRI programme demands a broad, context-sensitive knowledge of HPE as well as excellent teaching and research skills to run such programmes pg 8

Out of a total of 102 fellows, 35.3 per cent participated actively in the online discussion, which exceeded previous typical percentages of participation; there were 145 individual contributions to the discussions over 30 days. The discussion helped to clarify concepts related to CBME, and generated many themes such as the training of students (subject matter or clinical areas that can be covered through communitybased rotations), benefits to communities, the role of health managers, institution policy and procedural implications, the role of regulatory bodies, cost and the possible ethical mandate of CBME in developing countries. Moreover, 10 models of CBME from various countries. The active participation and high level of engagement while eliciting useful information about CBME also had an impact on subsequent online discussions on the list server. role-playing was picked up and used in the virtual session discussions by the fellows in one of FAIMER 's four regional institutes	Role-playing can be used effectively with health professions faculty members in the online environment, as it encourages distributed participation among a highly diverse group of international participants. Keeping a provocative aspect in the role-play scenario is useful to keep the participants engaged and the level of interest high. Improvements in the future might be to add intentional breaks from the role-playing for specific reflection on the discussion, which challenges participants to think even more deeply about the facts as they emerge P35

concepts related to CBME, and generated many themes such as the training of students (subject matter or clinical areas that can be covered through communitybased rotations), benefits to communities, the role of health managers, institution policy and procedural implications, the role of regulatory bodies, cost and the possible ethical mandate of CBME in developing countries. Moreover, 10 models of CBME from various countries (including Brazil, Egypt, Ethiopia, India, Nigeria, Nepal, Philippines, Pakistan and South Africa) were shared by the fellows. At the end, a summary of the whole discussion was shared with the list server group in the form of a comprehensive report. The active participation and high level of engagement while eliciting useful information about CBME also had an impact on subsequent online discussions on the list server. Another group	Keeping a provocative aspect in the role-play scenario is useful to keep the participants engaged and the level of interest high. Improvements in the future might be to add intentional breaks from the role-playing for specific reflection on the discussion, which challenges participants to think even more deeply about the facts as they emerge. As suggested by Ogilvie and Douglas, 'e-learning, when utilising a thoughtful design, can promote reflective dialogue and an understanding of theory'. (pp5-6)
The active participation and high level of engagement while eliciting useful information about CBME pg 34	Role-playing can be used effectively with health profes-sions faculty members in the online environment, as it encour-ages distributed participation among a highly diverse group of international participants. Keeping a provocative aspect in the role-play scenario is useful to keep the participants engaged and the level of interest high. pg 35

In contrast, our study demonstrated a varied response with lack of support regarding infrastructure regarding providing classroom environment in terms of ambience, furniture and internet provision. P933	many issues in learner’s presence linked with motivation and selfregulation… The cognitive presence in our study had problems in both the inquiry process as well as in collaboration with others. Physical fatigue during the contact sessions accounted for many of the issues that these students raised including the long working hours which caused students difficulty in exploration, construction, resolution, and understanding through collaboration and inquiry... Content organization plays a key role in the smooth experience in any educational program P932... institutions using blended learning programs that such programs should be able to enhance pedagogy, increase flexibility and access for students as well as provide a cost-effective resource usage.P933

learner's<br>presence linked with motivation and selfregulation. (p4)<br><br>There were students who found problems in<br>developing autonomy and becoming self-directed<br>learners. (p4)<br><br>Much blame was given to the previous traditional<br>teacher-led spoon fed learning approach which was<br>difficult to adapt. (p4)<br><br>Moreover, there were instances when the students felt lack of support from the<br>teachers in terms of their availability and timely<br>feedback provision. (p4)<br><br>The cognitive presence in our study had problems<br>in both the inquiry process as well as in collaboration<br>with others. Physical fatigue during	student's<br>responsibility also increases with demanding<br>self-regulation and self-directed learners. Proper<br>support, timely feedback and easy availability of<br>the facilitators can facilitate students in adopting to<br>challenging shift of traditional learning to blended<br>learning. The Facilitators play a myriad of roles<br>in blended programs, from instructor to mentor<br>and by providing timely feedback, and being<br>approachable, they can facilitate quality learning.<br>Institutional support can guarantee a smooth<br>educational experience for these students. The<br>study suggests that regular evaluation of blended<br>programs should be in practice to identify needs of<br>the stakeholders including students.
Students of MHPE are challenged with variety of issues in blended learning program relating to self-regulation, heavy cognitive load with engagement, social interaction especially with facilitators and managing group dynamics pg 929	The study suggests that regular evaluation of blended programs should be in practice to identify needs of the stakeholders including students. By answering to the students' needs and problems, improvements in students' experience can be ensured. Pg 933

There have been about a hundred major activities each year and many minor ones; participation in these activities has been good, with a total of 3193 conversations recorded as of April 08, 2018. About 20 publications in indexed journals have arisen through eJCIndia activities. P96	Capacity building in medical research is an important long-term goal for India and other developing countries. We believe that the successes of eJCIndia can be replicated in other medical disciplines in the country and suggest our effort as a model on which other initiatives in our and other medical disciplines can be launched at state, zonal, and even national levels in the country. The experiences and successes of eJCIndia can also be replicated in other developing countries for more efficient manpower development and capacity building in academic medicine. The availability of active, committed, competent, and experienced senior members for the provision of guidance, and a competent and efficient moderator, is a key to success. P96

in drafting a response to the PACECAR study were given inputs on improving their writing style. These discussions helped the participants improve analytical as well as writing skills. (p4)<br><br>Members of eJCIndia enthusiastically participated in the activity. Fourteen members working in academic institutions in different parts of the country volunteered to be a part of a multicenter project that would be conducted in several phases. The project protocol has been designed and is currently being reviewed and revised by the members before its submission to respective Institutional Ethics Committees for approval. (p4)<br><br>The fact that eJCIndia is 3 years old and still active and growing is itself an index of its success. There have been about a hundred major activities each year and many minor ones; participation in these activities has been good, with a total of 3193	Capacity building in medical research is an important long-term goal for India and other developing countries. We believe that the successes of eJCIndia can be replicated in other medical disciplines in the country and suggest our effort as a model on which other initiatives in our and other medical disciplines can be launched at state, zonal, and even national levels in the country. The experiences and successes of eJCIndia can also be replicated in other developing countries for more efficient manpower development and capacity building in academic medicine. The availability of active, committed, competent, and experienced senior members for the provision of guidance, and a competent and efficient moderator, is a key to success. (p4)
eJCIndia is 3 years old and still active and growing is itself an index of its success. There have been about a hundred major activities each year and many minor ones; participation in these ac-tivities has been good, with a total of 3193 conversations recorded as of April 08, 2018. About 20 publications in indexed journals have arisen through eJCIndia activities. pg 96	The availability of active, committed, competent, and ex-perienced senior members for the provision of guidance, and a competent and e?cient moderator, is a key to success. Pg 96

The workshops enhanced the faculty and student skills in using the technologies; however, they also served as a means to engage faculty and students in conversations about their own specific instructional needs. S103 encouraged faculty engagement S103 faculty realize the benefits of the adopted instructional technologies S103 The online master of health sciences program has managed to increase the number of streams offered from one in 2011 to five in 2014, indicating that uptake is on the increase. S104	enhanced eLearning faculty development program seems to be needed to address the challenges we have faced so far, specifically for faculty ICT literacy skills S104 Faculty engagement was the one component that all five schools reported a need to enhance S105
enhanced the faculty and student skills in<br>using the technologies; however, they also<br>served as a means to engage faculty and<br>students in conversations about their own<br>specific instructional needs. (p2)<br><br>Implementing LCMS+ as a hub<br>for eLearning not only improved<br>communications and organization<br>within the college but also encouraged<br>faculty engagement—for example,<br>other faculties and programs at the<br>college have asked to also use<br>LCMS+.<br>Although encouraging, this unintended<br>development points to the need to<br>make sure that implemented processes<br>continue to be efficient as more<br>departments seek to use them. (p2)<br><br>Besides being a method to distribute	degrees<br>of infrastructure and ICT expertise<br>within each school, faculty resistance to<br>committing more time to the development<br>of course content, and the absence<br>of institutional eLearning policies. A<br>consortium-level eLearning strategic plan<br>is under development to address these<br>challenges and operationalize the rollout<br>of the eLearning program to all medical<br>schools in the consortium. (p2)<br><br>However, for<br>sustainability of our eLearning program,<br>it is also important that institutional<br>leaders engage in discussions with key<br>government decision makers around<br>faculty incentives to increase adoption<br>and utilization of the eLearning
All five MEPI schools reported	Recognizing that faculty engagement

70 000 text messages were sent at a cost of 34 cents per message… 86% enjoyed and learned from the weekly text messages, 72% believed that the messages improved their clinical practice, and 68% regularly shared and discussed the messages with their colleagues. A total of 68% had access to the internet (56% in the public and 80% in the private sector), but only half used the facility. Most (98%) thought that the PEP books would help in their clinical practice, and 84% had<br>already purchased the books or intended to do so. All respondents wanted to receive further text messages on other important topics	text messages via personal cell phones<br>were well received by South African midwives; the information was widely shared with colleagues and was believed to improve learning and patient care. The messages often formed the basis of discussions and student teaching. The lack of access to the Internet, and failure to use this facility to obtain additional information, indicated that internet-based distance education still has limitations, especially in the public sector.... The use of cell phone text messaging promises to provide cost-effective learning opportunities, and improve a wide range of clinical services, such as the management of HIV-infected children and adults P101
The use of cell phone text messaging promises<br>to provide cost-effective learning opportunities, and improve a wide range of clinical services, such as the management of HIV-infected children and adults. (p2)	This survey demonstrated that text messages via personal cell phones were well received by South African midwives; the information was widely shared with colleagues and was believed to improve learning and patient care. The messages often formed the basis of discussions and student teaching. The lack of access to the Internet, and failure to use this facility to obtain additional information, indicated that internet-based distance education still has limitations, especially in the public sector. (p2)<br><br>Ongoing training and support ensure that students and faculty realize the benefits of the adopted instructional technologies. (p2)

This survey demonstrated that text messages via personal cell phones were well received by South African midwives; the information was widely shared with colleagues and was believed to improve learning and patient care. The messages often formed the basis of discussions and student teaching. pg 2	The use of cell phone text messaging promises to provide cost-effective learning opportunities, and improve a wide range of clinical services, such as the management of HIV-infected children and adults. Pg 2

Evaluation Framework	Evaluation Process
None	pre-validated questionnaire
Not described	satisfaction questionnaire, and some
Faculty member satisfaction pg 535	Faculty member satisfaction was measured using a pre-validated questionnaire pg 535

Community of inquiry and adaptation of Rourke et al categorization (3)	Emails were categorized as those that reflected cognitive presence, teacher presence, social presence and administrative emails<br>using a modification of the Community of Inquiry model (3) We also identified<br>three main aspects of social presence within emails: effective communication, open communication and group cohesion9. We<br>further categorized social responses into affective (dealing with values, beliefs, feelings, and emotions), cohesive (dealing with<br>group presence and commitment) and interactive (building up social bonds) (3)
Community of Inquiry model<br><br>Social presence<br><br>We further categorized social responses into affective (dealing with values, beliefs, feelings, and emotions), cohesive (dealing with group presence and commitment) and interactive (building up social bonds) according to Rourke et al.'s categorization (p2)	that reflected cognitive presence, teacher presence, social presence and administrative emails<br>using a modification of the Community of Inquiry model which defines cognitive presence as the exploration, construction,<br>resolution and confirmation of understanding through collaboration and reflection in a community of inquiry. Teacher presence<br>included emails with direct instruction to learners and learning resources. Resources included references to relevant journal articles, internet resources and other information exchanged across the listserv. Administrative emails were those that specified<br>deadlines for responses and emails sent by the moderators and faculty to streamline the discussion when it drifted off-track. Social<br>presence is described as the ability to project one's self and establish personal and purposeful relationships. We also identified<br>three main aspects of social presence

modification of the Community of Inquiry model which defines cognitive presence as the exploration, construction, resolution and confirmation of understanding through collaboration and reflection in a community of inquiry. pg 3	analyzed the number and content of emails exchanged during the month. Emails were categorized as those that reflected cognitive presence, teacher presence, social presence and administrative emails Pg 3
None	We stored the text messages of all e-mails which were exchanged during the discussion period. The e-mail responses satisfy the criterion of ‘low inference descriptors’ as participants do their own transcribing. During listserv communication, fellows contributed pieces of information to the data analysis discussion. In the first two weeks, 93 e-mails were exchanged, generating 24 pages of information (9,739 words) on quantitative data analysis. In the latter two weeks, 61 mails were exchanged resulting in 19 pages of information (7,810 words) on qualitative analysis. The responses for the initial needs assessment survey were also quantified. For e-mails relating to discussion, a manual content analysis was done to identify and retain the participants’ collective learning. Descriptive categories were formed from the contents of e-mails to characterize understanding of the fellows. The e-mail responses had characteristics of both speech and writing. Hence, the units of analysis were sentences and paragraphs

Not described	mails which were exchanged during the discussion period. The e-mail responses satisfy the criterion of 'low inference descriptors' as participants do their own transcribing8. During listserv communication, fellows contributed pieces of information to the data analysis discussion. In the first two weeks, 93 e-mails were exchanged, generating 24 pages of information (9,739 words) on quantitative data analysis. In the latter two weeks, 61 mails were exchanged resulting in 19 pages of information (7,810 words) on qualitative analysis. The responses for the initial needs assessment survey were also quantified. For e-mails relating to discussion, a manual content analysis was done to identify and retain the participants' collective learning9. Descriptive categories were formed from the contents of e-mails to characterize understanding of the fellows. The e-mail responses had characteristics of
a simple non-hierarchical typology which represents conceptions of the phenomenon by the fellows pg 3	stored the text messages of all e-mails which were exchanged pg 3 a manual content analysis was done to identify and retain the participants' collective leaening pg 3

Kirkpatrick model p4	a retrospective archival research design. SAFRI records and documents were reviewed to evaluate the fellowship programme as it relates to the development of HPE scholarship capacity in Africa. P3
Kirkpatrick model of evaluation (p3)	At the beginning of each residential
Kirkpatrick's evaluation framework pg 3	retrospective archival research design. SAFRI records and documents were reviewed to evaluate the fellowship programme as it re-lates to the development of HPE scholarship capacity in Africa. Pg 3

principles of adult learning, online learning and role-playing (Combined) p34	None

Not described	Not described
impact on subsequent online discussions on the list server. Pg 34	This re-use of the learning strategy by other fellows (health professions faculty members) is probably the best evidence that this group ?nds role-playing to be effective pg 34

Revised Garrison Community of Inquiry framework p930	individual, semi-structured audio-recorded, telephonic interviews. Two sets of six open-ended questions, each for students and facilitators ... triangulation was achieved by obtaining 902 students’ reflective essays from one university’s archival record after approval in order to check the consistency of findings... The interviews and essays were organized in Atlas-ti software for analysis. We adopted the Framework method of analysis with priori coding, using the Revised Garrison Community of Inquiry framework P930

Framework method of analysis with priori coding, using the Revised Garrison Community of Inquiry framework (p2)	semi-structured audio-recorded, telephonic interviews. Two sets of six open-ended questions, each for students and facilitators, were designed on the basis of Revised Garrison Community of Inquiry framework, which has been developed as a means to investigate effective online and blended learning environments in higher education.8 After pilot testing, the participants were approached, and informed consent was taken. The interviews were transcribed in English language and sent back to participants for member checking. Data saturation started occurring at the fourteenth student interview. However, four more interviews were recorded to countercheck the recurrence of codes in data.
Revised Garrison Community of In-quiry framework pg 930	individ-ual, semi-structured audio-recorded, telephonic interviews. Two sets of six open-ended questions,...The interviews were transcribed in English language and sent back to participants for member checking. Data source triangulation was achieved by obtaining 902 students' reflective essays from one university's archival record pg 930

None	None

Not described	Not described
head counts are all that is possible. Pg 96	e?orts are continuing, participation is good, and indices of success, such as participation, collaborations, and publications, suggest that the initiative enjoys reasonable success pg 96.

None	None
Not described	Not described
Zimbabwe: increased adoption of ICT	Zimbabwe: usage data

Survey	50 recipients were randomly identified:<br>25 in the public and private sectors, respectively. These midwives<br>were contacted by an independent consultant, and asked to answer 7 short questions; each question had 3 possible answers ranging from positive to negative P101
Not described	At the end of the project, 50 recipients were randomly identified:<br>25 in the public and private sectors, respectively. These midwives<br>were contacted by an independent consultant, and asked to answer 7 short questions; each question had 3 possible answers ranging from positive to negative (Table I).<br>Additional comments were recorded. (p2)

7 short questions; each question had 3 possible answers ranging from positive to negative pg2	50 recipients were randomly identified: 25 in the public and private sectors, respectively. These midwives were contacted by an independent consultant, and asked to answer 7 short questions; each question had 3 possible answers ranging from positive to negative (Table I). Additional comments were recorded. pg2

Comment
Single-page document

Quotations not copied

Quotations not copied

I just wanted to note that although
